# Pan-Chloroplast Genomes Reveal the Accession-Specific Marker for *Gastrodia elata* f. *glauca*

**DOI:** 10.3390/ijms252111603

**Published:** 2024-10-29

**Authors:** Jiaxue Li, Daichuan Pan, Junfei Wang, Xu Zeng, Shunxing Guo

**Affiliations:** State Key Laboratory of Bioactive Substance and Function of Natural Medicines, Institute of Medicinal Plant Development, Chinese Academy of Medical Sciences and Peking Union Medical College, Beijing 100193, China; 17749967508@163.com (J.L.); pandaichuan@126.com (D.P.); wjunfei2000@126.com (J.W.)

**Keywords:** chloroplast genomics, molecular marker, *Gastrodia elata*, genetic variation

## Abstract

*Gastrodia elata* rhizomes have been applied as traditional medicinal materials for thousands of years. In China, *G. elata* f. *elata* (red flower and stem, Ge), *G. elata* f. *viridis* (green, Gv), and *G. elata* f. *glauca* (black, Gg) represent the primary cultivars in artificial cultivation. Although the annual output of *G. elata* amounts to 150,000 tons, only 20% is Gg. The long production period, low yield, and high quality of Gg led to its extremely high market prices. However, an effective method to identify this crude drug based solely on its morphological or chemical characteristics is lacking. In this study, the complete chloroplast genomes of three *G. elata* variants were sequenced using the Illumina HiSeq 2500 platform. Another 21 chloroplast genomes from *Gastrodia* species, which have published in previous reports, were combined and analyzed together. Our results showed that larger genomic sizes, fewer long tandem repeats, and more simple sequence repeats were the major features of the Gg chloroplast genomes. Phylogenetic analysis showed that the Gg samples were separately clustered in a subclade. Moreover, an accession-specific marker was successfully developed and validated for distinguishing additional rhizome samples. Our study provides investigations of the taxonomic relationships of *Gastrodia* species. The molecular marker will be useful for differentiating *Gastrodia* products on the market.

## 1. Introduction

*Gastrodia elata* Blume, a myco-heterotrophic herb in the Orchidaceae family, is widely distributed across South and East Asia, including China, Japan, Korea, Nepal, Bhutan, and India [1]. This orchid possesses an underground rhizome traditionally utilized in oriental medicine. Over 200 compounds have been identified in *G. elata*. These compounds have been categorized into seven primary groups based on the core structure: aromatic compounds, steroids, furans, organic acids, polysaccharides and their glycosides, amino acids, and other compounds including sulfur and nitrogen derivatives [2]. Meanwhile, the neuroprotective properties of both the extract and individual monomers of *G. elata* have been confirmed via in vitro and in vivo studies [3,4], demonstrating significant pharmacological activity against numerous central nervous system disorders, such as epilepsy and Alzheimer’s disease [5]. Furthermore, this herb has exhibited a range of biological activities, including cognitive enhancement, antiepileptic, blood pressure reduction, hepatoprotective, antitumor activity, and immune system enhancement properties [6].

According to the record from the *Flora of China*, *G. elata* species contain five variants, including *G. elata* f. *elata*, *G. elata* f. *viridis*, *G. elata* f. *glauca*, *G. elata* f. *flavida*, and *G. elata* f. *alba* [7]. These variants exhibit distinct differences in flower and stem coloration, as well as tuber shape. *G. elata* f. *elata* (Ge, red flower, stem, and capsule), *G. elata* f. *viridis* (Gv, green), and *G. elata* f. *glauca* (Gg, black) represent the primary cultivars in artificial cultivation [2], predominantly grown in the provinces of Yunnan, Guizhou, Sichuan, Hubei, Shannxi, and Anhui [8]. The recent studies showed that the three main variants (Ge, Gv, and Gg) differ in their morphological characters, chemical compositions, and therapeutic impacts on diseases [2,9]. In general, Gg is mainly distributed in mountainous areas over 1600 m in elevation, while Ge and Gv are grown at an elevation of 600–1200 m. In addition, the longer production period, lower yield, and higher quality of Gg have led to its much higher value than those of Ge and Gv, with the price difference being a multiple of five in the market. However, it is difficult to distinguish between these variants based solely on their rhizomes, especially after processing, which are used for medicinal purposes. An effective method to identify this crude drug is still lacking.

With the development of high-throughput sequencing technology, molecular markers based on whole-genome sequences have been widely used in the genetic diversity analysis and germplasm identification of various economic crops. Recent developments in DNA marker technology, particularly those derived from chloroplast genomes, have markedly advanced phylogeny analyses, biodiversity assessments, and plant identification. Single-nucleotide polymorphisms (SNPs) and insertion deletion mutations (Indels) in genomic sequences have been globally accepted for species identification [10,11]. For instance, one study utilized the SNPs and Indels found in the intergenic regions of plastomes to evaluate the taxonomic relationships and develop molecular markers for medicinal *Alpinia* species [12]. A similar study implemented an allele-specific PCR (AS-PCR) technique for species-specific molecular diagnosis to differentiate between the green and purple varieties of purple tulsi based on the SNPs and Indels in the chloroplast genome [13]. The chloroplast genome harbors a diverse array of genes that are closely associated with photosynthesis and evolutionary processes [14,15]. This genome provides a robust framework for phylogenetic analysis and supports the domestication and enhancement of plant species [16]. The chloroplast genome, which is maternally inherited and haploid, features a highly conserved genomic structure and gene content [17]. In contrast, the non-coding regions exhibit rapid evolution due to nucleotide substitutions and the accumulation of insertion and deletion events, making it particularly suitable for studies on population genetic structure, historical population dynamics, and interspecific or subspecific comparisons [18]. Additionally, the high copy number of the chloroplast genome facilitates DNA extraction and the development of molecular markers for species identification [19].

The whole genome and transcriptome of *G. elata* have been published, and a large amount of genetic information has been revealed [20,21]. The previous studies found that numerous SNPs and simple sequence repeats (SSRs) could be used to distinguish *G. elata* variants [22]. In this study, 13 *G. elata* chloroplast genomes were used to develop molecular markers for variant identification. We sequenced the chloroplast genomes of three *G. elata* variants (Ge, Gv, and Gg) from the planting base. Three sets of raw sequencing data for *G. elata* obtained from the NCBI Sequence Read Archive (SRA) database were incorporated and assembled. In addition, another seven chloroplast genomes from *G. elata* and eleven chloroplast genomes from *Gastrodia* species (downloaded from the GenBank database) were analyzed together. The accession-specific marker within the hypervariable region of the chloroplast genome was explored to distinguish additional *G. elata* variant samples from the main producing areas in Sichuan, Guizhou, Yunnan, Shannxi, and Hubei. Our study of *G. elata* variant chloroplast genomes could provide a differentiating method for distinguishing true or fake *G. elata* f. *glauca*, an abundant data repository for *G. elata*, and the taxonomic relationships between *Gastrodia* species.

## 2. Results

### 2.1. Characteristics of G. elata Chloroplast Genomes

**Figure 1:**
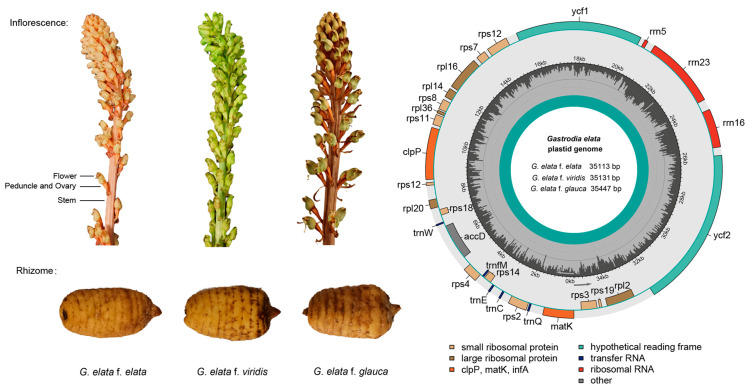
*Gastrodia elata* variant sample (inflorescence and rhizome) and chloroplast genome map (outer circle shows gene names, and dark grey bar on inner circle indicates GC content).

Similar to the previous studies, the *G. elata* chloroplast genome possessed a closed, circular, but not typical quadripartite structure. As shown in Figure 1, the chloroplast genomes of the *G. elata* variants lacked the typical large and small single-copy (LSC and SSC) and inverted repeat (IR) regions with twenty protein-coding genes (*rpl2*, *rpl14*, *rpl16*, *rpl20*, *rpl36*, *rps2*, *rps3*, *rps4*, *rps7*, *rps8*, *rps11*, *rps12*, *rps14*, *rps18*, *rps19*, *accD*, *clpP*, *matK*, *ycf1*, and *ycf2*), three rRNA genes (*rrn5*, *rrn16S*, and *rrn23*), and five tRNA genes (*trnC*-GCA, *trnE*-UUC, *trnfM*-CAU, *trnQ*-UUG, and *trnW*-CCA). Among them, *clpP* have two introns; *rps12*, *rpl16* and *rpl2* have one intron; and the rest have no introns. Numerous genes related to photosynthesis were lost, including photosystem I (*psa*), photosystem II (*psb*), the cytochrome b/f complex (*pet*), and ATP synthase (*atpA*). Based on the statistical t-test results, the chloroplast genomes of Gg (35,371 ± 66 bp) were, on average, statistically longer than those of Ge (35,158 ± 61 bp, *p* < 0.05) and Gv (35,134 ± 30 bp, *p* < 0.05) (Table 1). Except for *G. angusta* (36,812 bp), most of the *Gastrodia* species chloroplast genomes ranged in size from 29,696 to 31,896 bp and were smaller than the *G. elata* variants. The overall GC contents of the *Gastrodia* chloroplast genomes were 24.8–26.8%.

### 2.2. Codon Usage Analysis

**Figure 2:**
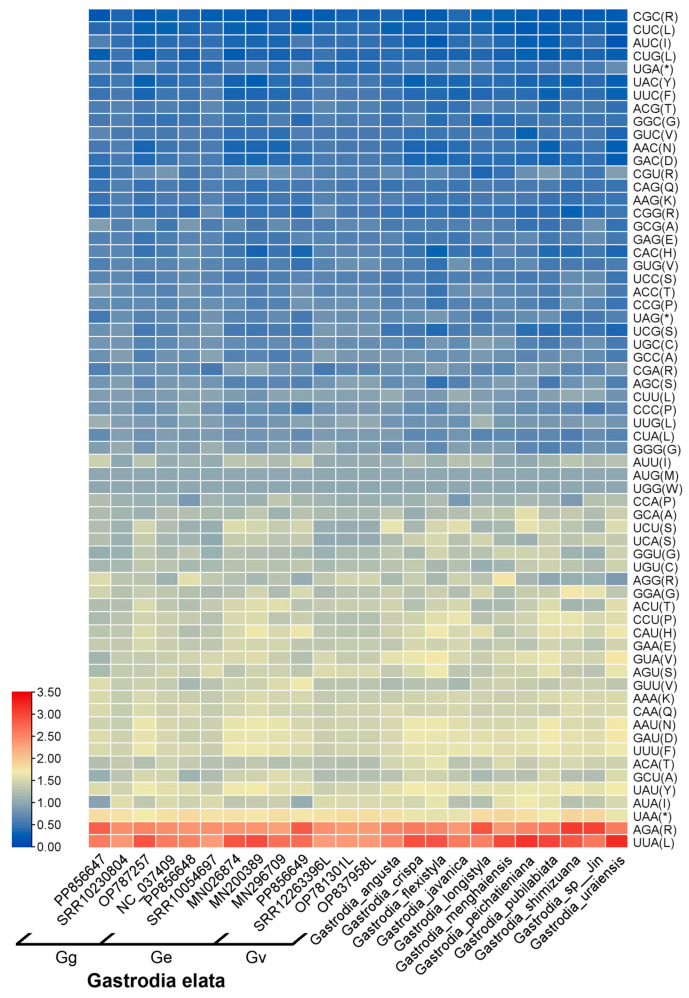
Heat map shows the differences in synonymous codon usage among *Gastrodia* species based on RSCU values. * represents termination codon.

The combined effects of gene selection, mutation, and other factors during long-term biological evolution have led to differences in the codon usage frequency between most organisms, with each species exhibiting unique codon usage preferences [23]. The chloroplast genomes of 13 *G. elata* variants and 11 other *Gastrodia* species encoded 8774–9751 and 8042–9728 codons (Table S1). Like other *Gastrodia* species, in terms of high-frequency codons, the *G. elata* variants chloroplast genomes exhibited 26–29 codons with relative synonymous codon usage (RSCU) values greater than one, indicating their frequent usage (Figure 2). In general, the codon for arginine (CGC) had the lowest usage frequency, with RSCU values of 0.21–0.36 in *Gastrodia*. Since methionine (Met) and tryptophan (Trp) have only one codon each (AUG and UGG, respectively), there was no codon usage preference for these amino acids. In detail, CUU had a high frequency in four other *Gastrodia* species, but not in *G. elata*. Conversely, only three *G. elata* variants had RSCU values greater than one for GGG. Based on statistical testing between the *G. elata* variants, Gg use fewer GUA codons and more AGA codons than Ge (*p* < 0.05), Gg use fewer CCG codons and CUU codons than Gv (*p* < 0.05), there was no significant difference between Ge and Gv. Additionally, there were 30 low-frequency codons, most of which ended in G or C. Our results suggest that the chloroplast genomes of *G. elata* preferred the codons ending in A and U, which is common in most chloroplast genomes of angiosperms [24].

### 2.3. Long Tandem Repeats and SSR Polymorphism

A total of 1018 long tandem repeats were detected in the 24 *Gastrodia* chloroplast genomes, with *G. menghaiensis* having the highest count of 124 repeat sequences (Table 2). A total of 6.78% of the repeat sequences in *Gastrodia* chloroplast genomes were more than 50 bp in length. These chloroplast genomes primarily contained four types of repeats: forward, inverse, complement, and palindromic. The lengths of these repeat sequences ranged from 30 to 267 bp (Table S2). Overall, the *G. elata* chloroplast genomes contained fewer repeat sequences than the other *Gastrodia* species, especially a lack of inverse and complement repeats. The Gg chloroplast genomes had the lowest count of fifteen tandem repeats with the lengths ranging from 30 to 83 bp (Table S2), while the Ge and Gv chloroplast genomes possessed two or three additional palindromic repeats.

SSRs are a type of microsatellite sequence composed of motifs ranging from 1 to 6 bp in length as repeating units. They can be distributed across various positions within the entire genome and are widely used in species identification, population genetics, and phylogenetic polymorphism studies. With 1010 SSRs in total, six types (mono-, di-, tri-, tetra-, penta-, and hexa-nucleotide repeat sequences) were identified in the chloroplast genomes of *Gastrodia* species. Mono-nucleotide repeat sequences were the most abundant (33.3–67.6%), and all were A, T repeats. A total of five types of SSRs, numbered 37–45, were found in the *G. elata* chloroplast. Moreover, hexa-nucleotide SSRs were only detected in a few *Gastrodia* species, such as *G. angusta* and *G. longistyla* (Table S3). Among them, some mono-nucleotide repeat (T), di-nucleotide repeat (AT or TA), tri-nucleotide repeat (ATA), tetra-nucleotide repeat (TTAT or TATT), and penta-nucleotide repeat (TAAAA) sequences were more abundant in the Gg chloroplast genomes than the Ge and Gv chloroplast genomes. In contrast, some tri-(AAT or ATT), tetra-(TAAA) and penta-nucleotide (AATAT) repeats were only found in the Ge and Gv chloroplast genomes.

### 2.4. Phylogenetic Analysis

**Figure 3:**
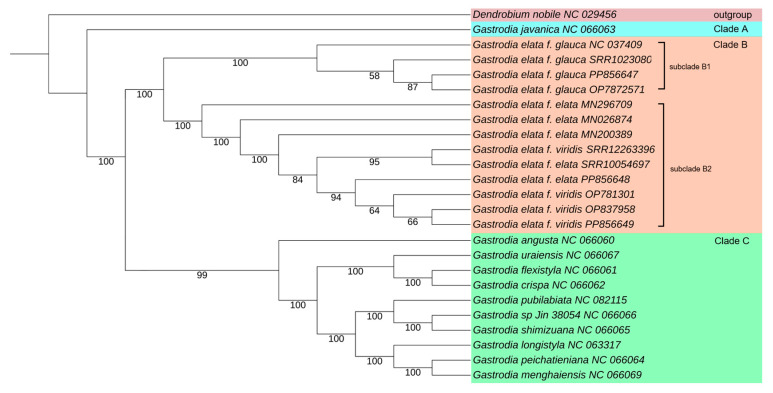
Maximum likelihood phylogenetic tree of *Gastrodia* species based on 18 shared chloroplast protein-coding sequences. Bootstrap scores were calculated from 1000 replicates.

The plant chloroplast genomes have revealed greatly divergent evolutionary trajectories [25]. A total of 18 protein-coding genes from the 24 *Gastrodia* chloroplast genomes and *D. nobile* (as the outgroup) were used for phylogenetic analysis using the maximum likelihood method. As shown in Figure 3, *G. javanica* was differentiated earliest from the main clade of the *Gastrodia* genus, serving as the sister species to other *Gastrodia* species (bootstrap score, BS = 100%). All of *G. elata* were clustered in clade B with high bootstrap support values (BS = 100%). In detail, the variants of *G. elata* were distributed among two main subclades. The first subclade B1 contained only Gg, and the second subclade, B2, contained both Ge and Gv. Most of the branches exhibited high bootstrap scores, signifying the phylogenetic tree’s strong reliability.

### 2.5. Nucleotide Diversity

**Figure 4:**
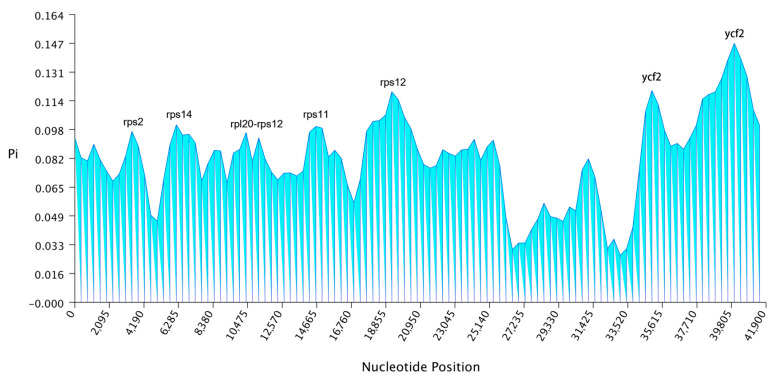
Nucleotide diversity (Pi) across the chloroplast genomes of *Gastrodia* species.

The nucleotide polymorphism (Pi) values of each fragment in the *Gastrodia* chloroplast genomes were calculated. The average Pi value of all the fragments was 0.081908 (Table S4). Many regions exhibited nucleotide polymorphism. The fragment with the most nucleotide polymorphisms in the genome was the *ycf2* gene, with a Pi value of 0.14716. In addition, six divergent regions showed remarkably high Pi values (*rps2*, *rps14*, *rpl20-rps12*, *rps11*, *rps12-rps12*, and *ycf2*), which can serve as potential molecular markers for the species identification of the *Gastrodia* genus (Figure 4).

### 2.6. Development and Validation of Molecular Markers

**Figure 5:**
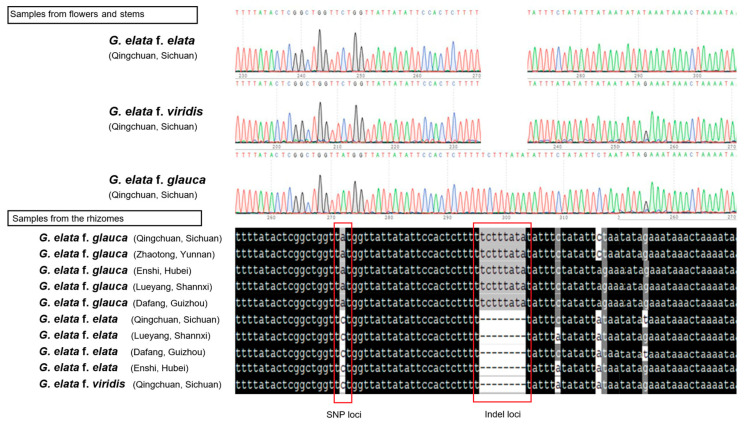
The development and validation of the accession-specific marker for the *G. elata* variants. The peak maps of the flower and stem samples (**up**) and the sequencing results of the rhizome samples (**down**) from each *G. elata* variant are shown.

Through counting, 469 SNPs were identified within the *G. elata* variants. Molecular markers were developed from the highly variable regions based on the comparative analysis of the chloroplast genomes to distinguish the *G. elata* variants. Hence, an accession-specific molecular marker located at the position *ycf2* was investigated. The PCR primers used to amplify were Ycf2-F: GTTTCATTACGTCCTC and Ycf2-R: TACGACCTTTACCACA. We identified one hypervariable region (one specific SNP loci and one Indel loci) that could differentiate the *G. elata* variants (Figure 5). A total of 10 rhizome samples of *G. elata* variants from the five main producing areas also demonstrated that Gg could be successfully differentiated from Ge and Gv using this molecular marker.

## 3. Discussion

As a fully heterotrophic plant without photosynthesis, *G. elata* chloroplast genomes are much smaller than those of autotrophic plants, such as *Hibiscus rosa-sinensis* (160,951 bp) and *Dodonaea viscosa* (159,375 bp) [26,27]. Numerous genes related to photosynthesis were not detected in the *G. elata* chloroplast genomes, including photosystem I, photosystem II, cytochrome b6f, cytochrome C6, ATP synthase, and Rubisco14. The remaining 28 genes in the *G. elata* chloroplast genomes are all associated with self-replication [28]. In this study, all the *G. elata* chloroplast genomes exhibited consistency in gene order and content, implying a highly conserved genome structure. As expected, the LSC, SSC, and IR regions were not distinct in the *G. elata* circular chloroplast genome. The previous studies suggested that IR region loss is very common in parasitic and saprophytic plants, and this has been partially verified in the *Epipogium* and *Sciaphila* genera [29,30]. Our results support the idea that *G. elata* chloroplast genomes could lose several functional genes and adapt heterotrophy at a later evolution stage [31].

Repetitive sequences play an important role in chloroplast genome research. Multiple-copy nucleic acid sequences have great significance for chromosome rearrangement and biological evolution. In our study, four kinds of long tandem repeats were identified in the *Gastrodia* genus. In particular, most of *G. elata* chloroplast genomes lost inverse and complement repeats. Moreover, Gg contained fewer palindromic repeats than Ge or Gv. Our results indicated that repetitive sequences exhibit variation within both the genus and the species. In total, these repeats can facilitate structural rearrangements and contribute to variations among the chloroplast genomes within a population.

SSRs, another type of repetitive sequence, are also widely distributed and highly variable in the genome. A previous study found that a high frequency of SSRs in the genome indicates a high mutation rate in that region, which could reflect the genetic diversity of the species [32]. The molecular markers developed from chloroplast genome SSRs play an important role in species identification and phylogenetic research. Here, the types and quantities of SSRs varied between the different species within the *Gastrodia* genus. Consistent with other species [14], mono-nucleotide repeats were the most common type of repeat in the *Gastrodia* chloroplast genome. Most of *Gastrodia* species exhibited a greater abundance of A/T mono-nucleotides, similar to many angiosperms [33]. This might be due to A/T transitions being easier than G/C transitions in plant chloroplast genomes. Meanwhile, among the *G. elata* variants, the number of SSRs for Gg differed from Ge and Gv, which indicates that SSRs could reflect the genetic diversity of *G. elata*.

The phylogenetic analysis of the *Gastrodia* genus had previously been performed based on morphological features and DNA sequences, such as internal transcribed spacer (ITS, in the nucleus) and protein-coding genes (*rbcL*, in the chloroplast genome) [34,35]. In our results, *G. javanica* was not clustered with any other *Gastrodia* species. This plant is mainly distributed in tropical regions, including Thailand, Malaysia, and the Philippines, unlike others that are distributed in temperate regions [36]. In clade C of our phylogenetic tree, ten *Gastrodia* species were grouped together and distinguished from each other with high bootstrap scores. *G. javanica*, *G. elata*, and *G. angusta* are all non-rooted plants, but possess well-developed tubers and bulbs. In contrast, the remaining nine *Gastrodia* species have developed root systems, but have small and dark tubers and bulbs. Notably, the *G. elata* variants in clade B showed that Gg was categorized into subclade B1, whereas Ge was clustered together with Gv in subclade B2. Phylogenetic analysis showed that *Gastrodia* classification might be related to the survival environments and root systems of plants.

Five *G. elata* variants are primarily distributed in China; Ge, Gv, and Gg represent the primary cultivars in artificial cultivation. In recent years, the annual output of fresh *G. elata* has amounted to 150,000 tons, 80% of which is Ge or Gv, and 20% is Gg. A recent study found that some phytometabolites were significantly higher in Gg, including parishins, vanilloloside, and gastrodin A/B [2]. Another study showed that the content of polysaccharides, such as total sugar and total polyphenol, were high in Gg, and differences were found in the in vitro antioxidant and hypoglycemic activities among the *G. elata* variants [9]. The longer production period, lower yield, and higher quality of Gg have resulted in its higher prices than those of Ge or Gv. Many fake and counterfeit products of Gg have appeared on the market. It is important to develop a reliable method to distinguish *G. elata* variants. However, the morphological differences between Ge, Gv, and Gg are mainly in the color of flowers, stems, and capsules. Moreover, medicinal rhizomes are usually sold after drying, making it hard to identify market samples. The early development of molecular markers had shortcomings, such as low-level polymorphism and instability [37]. In our study, the *G. elata* chloroplast genome was sequenced based on the flowers, capsules, and stems with doubtless color. Subsequently, we designed specific primer pairs based on chloroplast genome interspecific analysis. The target fragments were amplified in ten rhizome samples of *G. elata* variants from the five main producing areas. The sequencing of the PCR products showed that this accession-specific marker demonstrates great potential to identify Gg variants.

As obligate myco-heterotrophs lacking chlorophyll, *Gastrodia* chloroplast genomes are significantly degenerated and considerably shorter than those of typical angiosperms, making *Gastrodia* an ideal model for studying plastome evolution [38,39]. While some phylogenetic studies using chloroplast genomes of *Gastrodia* have been undertaken, a thorough investigation of the chloroplast genome structure and diversity at the population level is still needed. Moreover, a systematic study focusing on the molecular markers of more *G. elata* variants needs to be conducted.

## 4. Materials and Methods

### 4.1. Sample Collection, DNA Extraction, and Chloroplast Genome Sequencing

The inflorescences of Ge, Gv, and Gg, including the flowers, the stems, and a small number of capsules (postdevelopmental period for peduncle and ovary), were harvested for DNA extraction from a cultivation area located in Qingchuan County, Sichuan Province, China (104.80° E, 32.37° N, at an elevation of 1726.3 and 1217.4 m). Total DNA was extracted using a specialized plant genomic DNA kit (Tiangen Biotech, Beijing, China). The integrity and purity of the extracted DNA were assessed via 1% agarose gel electrophoresis, and its concentration was precisely quantified using a Nanodrop spectrophotometer 2000 (Thermo Fisher Scientific, Waltham, MA, USA). Subsequently, high-quality genomic DNA samples were fragmented to construct 300 bp paired-end libraries, which were then sequenced using the PE150 protocol on an Illumina HiSeq 2500 platform (Illumina, San Diego, USA), ensuring comprehensive genetic analysis.

### 4.2. Chloroplast Genome Assembly and Annotation

The three raw sequencing reads of *G. elata* (Ge, Gv, and Gg), along with another three raw reads of *G. elata* downloaded from the NCBI SRA database were assembled using GetOrganelle v1.64 with default parameters [40]. Annotation was conducted using the CPGAVAS2 web server (http://www.herbalgenomics.org/cpgavas2, accessed on 22 May 2024), and manual correction was performed using Apollo software v2.5.0 [41,42]. The chloroplast genomes of the samples were visualized using Chloroplot (https://irscope.shinyapps.io/Chloroplot/, accessed on 1 June 2024) [43]. All of the chloroplast genomes in our study were deposited in GenBank with accession numbers PP856647, PP856648, and PP856649, respectively. Three sets of raw sequencing data for *G. elata* obtained from the NCBI Sequence Read Archive (SRA) database were incorporated and assembled. Furthermore, the chloroplast genomes of seven *G. elata* (Ge, Gv, and Gg) and eleven *Gastrodia* species (*G. angusta*, *G. crispa*, *G. flexistyla*, *G. javanica*, *G. longistyla*, *G. menghaiensis*, *G. peichatieniana*, *G. pubilabiata*, *G. shimizuana*, *G. uraiensis*, and *Gastrodia* sp. Jin) were downloaded from the GenBank database, and subsequently analyzed. All 24 chloroplast genomes are listed in Table 1.

### 4.3. Repeat Element Analysis

Long tandem repeats were detected using Tandem Repeat Finder (https://tandem.bu.edu/trf/trf.html, accessed on 9 June 2024) [44] and REPuter software (https://bibiserv.cebitec.uni-bielefeld.de/reputer, accessed on 9 June 2024), with the settings adjusted to a minimum repeat size of 20 base pairs and a similarity threshold of over 90% between the repeats [45].

The identification of SSRs was carried out using MISA software (http://pgrc.ipk-gatersleben.de/misa/, accessed on 10 June 2024) [46], setting the minimum number of repeats at 10, 5, 4, 3, 3, and 3 for mono-, di-, tri-, tetra-, penta-, and hexa-nucleotides, respectively. The minimum interspersed distance required between two SSRs was set to 100 base pairs.

### 4.4. Phylogenetic Analysis

All the shared protein-coding sequences (CDSs) of the chloroplast genome, with *Dendrobium nobile* serving as the outgroup, were utilized to construct the phylogenetic relationships. The shared CDSs were extracted using PhyloSuite v1.2.2 [47], and subsequently aligned using MAFFT v7.471 [48]. These aligned sequences then formed the basis for maximum-likelihood (ML) phylogenetic analysis conducted in IQ-TREE v1.6.12 with a best-fit model (K3Pu + F + I) [49]. Bootstrap analysis was performed using 1000 replicates to assess the reliability of the phylogenetic tree.

### 4.5. Genomic Analysis and Nucleotide Variation Analysis

The GC content was calculated using Chloroplot [43], and codon usage was analyzed using MEGA X software v10.2.6 [50]. The codon usage bias was calculated using IBM SPSS Statistics v26 (SPSS Inc., Chicago, IL, USA) with the Wilcoxon rank sum test [51]. Nucleotide variability (Pi) was calculated using DnaSP v5.1 to compare nucleic acid diversities [52].

### 4.6. Development and Validation of Molecular Markers

The highly variable regions of the *G. elata* chloroplast genomes were investigated in detail to develop molecular markers that can discriminate them. PCR amplification primers were designed using the Primer3 program (http://bioinfo.ut.ee/primer3-0.4.0/, accessed on 1 July 2024). The primers sequences were compared with those of other species using the CBI Multiple Sequence Alignment Viewer v1.21.0 (max seq difference = 0.75) on the BLASTN website [53]. PCR was performed using a total volume of 25 μL containing 12.5 μL of 2× Taq PCR Master Mix, 0.4 μM of each primer, 1 μL of template DNA, and 9.5 μL of ddH_2_O. The PCR program was as follows: 3 min at 94 °C; for initial DNA denaturation, 35 cycles of 15 s at 95 °C, 20 s at 46 °C, and 30 s at 72 °C; and 5 min at 72 °C for final extension. The PCR products were visualized using 1.0% AGE polyacrylamide gel electrophoresis, and then whole sequences were obtained using Sanger sequencing with the same set of primers used for PCR amplification. Moreover, 10 samples of *G. elata* variants from the main producing areas in Sichuan, Guizhou, Yunnan, Shannxi, and Hubei were used to validate the accession-specific molecular marker.

## 5. Conclusions

In this study, the chloroplast genomes of 13 *G. elata* and 11 another *Gastrodia* species were analyzed together. Despite their similar genome structures and gene contents, their chloroplast genomes exhibited differences in repeat sequences and codon usage bias, which fully illustrated the relevance and distinction between these species. Phylogenetic analysis showed the taxonomic relationships between these *Gastrodia* species and supported the previous phylogeny. We developed an accession-specific marker for distinguishing the *G. elata* varieties and verified its effectiveness based on the highly variable regions. Our study ensures the efficacy and safety of *G. elata* medicinal products, laying the foundation for their further utilization. Simultaneously, *Gastrodia* is an ideal model for studying chloroplast genome evolution, which can provide new insights into the evolution of heterotrophic plants.

## Figures and Tables

**Table 1:** Summary of chloroplast genome features of *Gastrodia* species.

Species	GeneBank Accession	Size	GC%	Total Genes	Protein-Coding Genes	tRNA Genes	rRNA Genes
*Gastrodia elata* f. *glauca*	PP856647	35,447	26.7%	28	20	5	3
*Gastrodia elata* f. *glauca*	SRR10230804	35,328	26.8%	28	20	5	3
*Gastrodia elata* f. *glauca*	NC037409	35,304	26.8%	28	20	5	3
*Gastrodia elata* f. *glauca*	OP787257	35,407	26.7%	28	20	5	3
*Gastrodia elata* f. *elata*	PP856648	35,113	26.8%	28	20	5	3
*Gastrodia elata* f. *elata*	SRR10054697	35,178	26.7%	28	20	5	3
*Gastrodia elata* f. *elata*	MN026874	35,230	26.7%	28	20	5	3
*Gastrodia elata* f. *elata*	MN296709	35,193	26.7%	28	20	5	3
*Gastrodia elata* f. *elata*	MN200389	35,079	26.7%	28	20	5	3
*Gastrodia elata* f. *viridis*	PP856649	35,131	26.7%	28	20	5	3
*Gastrodia elata* f. *viridis*	SRR12263396	35,178	26.7%	28	20	5	3
*Gastrodia elata* f. *viridis*	OP837958	35,115	26.7%	28	20	5	3
*Gastrodia elata* f. *viridis*	OP781301	35,112	26.7%	28	20	5	3
*Gastrodia angusta*	NC066060	36,812	25.4%	29	20	5	4
*Gastrodia crispa*	NC066062	30,582	25.7%	28	19	5	4
*Gastrodia flexistyla*	NC066061	30,797	25.4%	28	19	5	4
*Gastrodia javanica*	NC066063	31,896	24.8%	27	19	4	4
*Gastrodia longistyla*	NC063317	30,464	26.8%	26	18	5	3
*Gastrodia menghaiensis*	NC066069	30,158	26.8%	28	19	5	4
*Gastrodia peichatieniana*	NC066064	29,696	25.9%	28	19	5	4
*Gastrodia pubilabiata*	NC082115	30,698	24.9%	25	19	3	3
*Gastrodia shimizuana*	NC066065	30,019	25.5%	28	19	5	4
*Gastrodia* sp. *Jin*	NC066066	29,944	25.8%	28	19	5	4
*Gastrodia uraiensis*	NC066067	30,746	24.9%	28	19	5	4

**Table 2:** The long tandem repeats in the chloroplast genomes of *Gastrodia* species.

Species	GeneBank Accession	Forward Repeats	Palindromic Repeats	Inverse Repeats	Complement Repeats	>50 bp	Total
*Gastrodia elata* f. *glauca*	PP856647	3	13	0	0	2	16
*Gastrodia elata* f. *glauca*	SRR10230804	2	13	0	0	2	15
*Gastrodia elata* f. *glauca*	NC037409	2	13	0	0	2	15
*Gastrodia elata* f. *glauca*	OP787257	3	12	0	0	2	15
*Gastrodia elata* f. *elata*	PP856648	2	15	0	0	3	17
*Gastrodia elata* f. *elata*	SRR10054697	3	16	1	0	2	20
*Gastrodia elata* f. *elata*	MN026874	2	13	0	0	2	15
*Gastrodia elata* f. *elata*	MN296709	2	14	0	0	3	16
*Gastrodia elata* f. *elata*	MN200389	2	14	0	0	3	16
*Gastrodia elata* f. *viridis*	PP856649	2	15	0	0	3	17
*Gastrodia elata* f. *viridis*	SRR12263396	3	16	1	0	2	20
*Gastrodia elata* f. *viridis*	OP837958	2	15	0	0	3	17
*Gastrodia elata* f. *viridis*	OP781301	2	15	0	0	3	17
*Gastrodia angusta*	NC066060	42	30	35	12	2	119
*Gastrodia crispa*	NC066062	42	30	35	12	1	119
*Gastrodia flexistyla*	NC066061	7	18	7	3	2	35
*Gastrodia javanica*	NC066063	16	15	14	7	1	52
*Gastrodia longistyla*	NC063317	47	14	38	8	7	107
*Gastrodia menghaiensis*	NC066069	47	22	51	4	11	124
*Gastrodia peichatieniana*	NC066064	14	19	20	1	5	54
*Gastrodia pubilabiata*	NC082115	31	35	33	18	4	117
*Gastrodia shimizuana*	NC066065	9	9	10	1	2	29
*Gastrodia* sp. *Jin*	NC066066	7	12	9	0	1	28
*Gastrodia uraiensis*	NC066067	3	12	2	1	1	18

## Data Availability

Data are contained within the article and Supplementary Materials.

## Supplementary Materials

The following supporting information can be downloaded at: https://www.mdpi.com/article/10.3390/ijms252111603/s1.

## References

[1] Tsai C.F., Huang C.L., Lin Y.L., Lee Y.C., Yang Y.C., Huang N.K. (2011). The neuroprotective effects of an extract of *Gastrodia elata*. J. Ethnopharmacol..

[2] Zeng X., Li J., Chen T., Li Y., Guo S. (2023). Global metabolic profile and multiple phytometabolites in the different varieties of *Gastrodia elata* Blume. Front. Plant Sci..

[3] Zhou B., Tan J., Zhang C., Wu Y. (2018). Neuroprotective effect of polysaccharides from *Gastrodia elata* Blume against corticosterone-induced apoptosis in PC12 cells via inhibition of the endoplasmic reticulum stress-mediated pathway. Mol. Med. Rep..

[4] Liu C.M., Tian Z.K., Zhang Y.J., Ming Q.L., Ma J.Q., Ji L.P. (2020). Effects of gastrodin against lead-induced brain injury in mice associated with the wnt/nrf2 pathway. Nutrients.

[5] Lu Z., Fu J., Wu G., Yang Z., Wu X., Wang D., You Z., Nie Z., Sheng Q. (2023). neuroprotection and mechanism of Gas-miR36-5p from *Gastrodia elata* in an Alzheimer’s disease model by regulating glycogen synthase kinase-3β. Int. J. Mol. Sci..

[6] Gong M., Lai F., Chen J.Z., Li X., Chen Y., He Y. (2024). Traditional uses, phytochemistry, pharmacology, applications, and quality control of *Gastrodia elata* Blume: A comprehensive review. J. Ethnopharmacol..

[7] Li Z., Bao Z., Huang H. (2011). Allozymic variation and genetic relationship among *Gastrodia elata* forms, a medicinal plant in China. Plant Sci. J..

[8] Hu J., Feng Y., Zhong H., Liu W., Tian X., Wang Y., Tan T., Hu Z., Liu Y. (2023). Impact of climate change on the geographical distribution and niche dynamics of *Gastrodia elata*. PeerJ.

[9] Ji N., Liu P., Zhang N., Yang S., Zhang M. (2022). Comparison on bioactivities and characteristics of polysaccharides from four varieties of *Gastrodia elata* Blume. Front. Chem..

[10] Kumar P., Gupta V.K., Misra A.K., Modi D.R., Pandey B.K. (2009). Potential of molecular markers in plant biotechnology. Plant Omics J..

[11] Kumar S., Banks T., Cloutier S. (2012). SNP discovery through next-generation sequencing and its applications. Int. J. Plant Genom..

[12] Yang H., Wang L., Chen H., Jiang M., Wu W., Liu S., Wang J., Liu C. (2021). Phylogenetic analysis and development of molecular markers for five medicinal *Alpinia* species based on complete plastome sequences. BMC Plant Biol..

[13] Balaji R., Parani M. (2024). Development of an allele-specific PCR (AS-PCR) method for identifying high-methyl eugenol-containing purple tulsi (*Ocimum tenuiflorum* L.) in market samples. Mol. Biol. Rep..

[14] Ren T., Yang Y., Zhou T., Liu Z. (2018). Comparative plastid genomes of *Primula* species: Sequence divergence and phylogenetic relationships. Int. J. Mol. Sci..

[15] Chong X., Li Y., Yan M., Wang Y., Li M., Zhou Y., Chen H., Lu X., Zhang F. (2022). Comparative chloroplast genome analysis of 10 *Ilex* species and the development of species-specific identification markers. Ind. Crops Prod..

[16] Cao S., Zhang H., Liu Y., Sun Y., Chen Z.J. (2024). Cytoplasmic genome contributions to domestication and improvement of modern maize. BMC Biol..

[17] Zhang H., Liu P., Zhang Y., Sun H., Wang Y., Gao Z., Liu X. (2024). Chloroplast genome of *Calamus tetradactylus* revealed rattan phylogeny. BMC Genome Data.

[18] Downie S.R., Katz-Downie D.S., Watson M.F. (2000). A phylogeny of the flowering plant family Apiaceae based on chloroplast DNA *rpl16* and *rpoC1* intron sequences: Towards a suprageneric classification of subfamily Apioideae. Am. J. Bot..

[19] Jung J., Kim K.H., Yang K., Bang K.H., Yang T.J. (2014). Practical application of DNA markers for high-throughput authentication of *Panax ginseng* and *Panax quinquefolius* from commercial ginseng products. J. Ginseng Res..

[20] Wang Y., Shahid M.Q., Ghouri F., Baloch F.S. (2020). De novo assembly and annotation of the juvenile tuber transcriptome of a *Gastrodia elata* hybrid by RNA sequencing: Detection of SSR markers. Biochem. Genet..

[21] Xu Y., Lei Y., Su Z., Zhao M., Zhang J., Shen G., Wang L., Li J., Qi J., Wu J. (2021). A chromosome-scale *Gastrodia elata* genome and large-scale comparative genomic analysis indicate convergent evolution by gene loss in mycoheterotrophic and parasitic plants. Plant J..

[22] Wang Y., Shahid M.Q., Ghouri F., Ercişli S., Baloch F.S. (2019). Development of EST-based SSR and SNP markers in *Gastrodia elata* (herbal medicine) by sequencing, de novo assembly and annotation of the transcriptome. 3 Biotech.

[23] Liu Q., Xue Q. (2005). Comparative studies on codon usage pattern of chloroplasts and their host nuclear genes in four plant species. J. Genet..

[24] Munyao J.N., Dong X., Yang J.X., Mbandi E.M., Wanga V.O., Oulo M.A., Saina J.K., Musili P.M., Hu G. (2020). Complete chloroplast genomes of *Chlorophytum comosum* and *Chlorophytum gallabatense*: Genome structures, comparative and phylogenetic analysis. Plants.

[25] Wang J., Kan S., Liao X., Zhou J., Tembrock L.R., Daniell H., Jin S., Wu Z. (2024). Plant organellar genomes: Much done, much more to do. Trends Plant Sci..

[26] Abdullaha M.F., Shahzadia I., Waseemb S., Mirzaa B., Ahmedb I., Waheed M.T. (2020). Chloroplast genome of *Hibiscus rosa-sinensis* (Malvaceae): Comparative analyses and identification of mutational hotspots. Genomics.

[27] Saina J.K., Gichira A.W., Li Z., Hu G., Wang Q., Liao K. (2018). The complete chloroplast genome sequence of *Dodonaea viscosa*: Comparative and phylogenetic analyses. Genetica.

[28] Xu X., Wang D. (2021). Comparative chloroplast genomics of *Corydalis species* (Papaveraceae): Evolutionary perspectives on their unusual large scale rearrangements. Front. Plant Sci..

[29] Schelkunov M.I., Shtratnikova V.Y., Nuraliev M.S., Selosse M.A., Penin A.A., Logacheva M.D. (2014). Exploring the limits for reduction of plastid genomes: A case study of the mycoheterotrophic orchids *Epipogium aphyllum* and *Epipogium roseum*. Genome Biol. Evol..

[30] Petersen G., Zervas A., Pedersen H.E., Seberg O. (2018). Genome reports: Contracted genes and dwarfed plastome in mycoheterotrophic *Sciaphila thaidanica* (Triuridaceae, Pandanales). Genome Biol. Evol..

[31] Yuan Y., Jin X., Liu J., Zhao X., Zhou J., Wang X., Wang D., Lai C., Xu W., Huang J. (2018). The *Gastrodia elata* genome provides insights into plant adaptation to heterotrophy. Nat. Commun..

[32] Cavalier-Smith T. (2002). Chloroplast evolution: Secondary symbiogenesis and multiple losses. Curr. Biol..

[33] Xie D.F., Yu Y., Zhou S., Yu H., Price M., Xie C., Deng Y., Chen J., Yu Y., Zhou S. (2019). Phylogeny of Chinese *Allium* species in section Daghestanica and adaptive evolution of *Allium* (Amaryllidaceae, Allioideae) species revealed by the chloroplast complete genome. Front. Plant Sci..

[34] Zhang J., Yu H., Li Z.H., Chen B.Q., Huang H., Zhang X.G., Lv G.H. (2024). Comparison of tuber characteristics and ITS sequence of four variants of medicinal *Gastrodia elata*. Chin. Herb. Med..

[35] Tang K. (2013). Research on DNA Barcoding and Medicinal Component Analysis of Zhaotong *Gastrodia elata*. Master’s Thesis.

[36] Wen Y.Y. (2023). Evolutionary Biology of Plastid Genomes of *Gastrodiea* (Orchidaceae). Master’s Thesis.

[37] Wang C.Y., Hou J., Zhou M.C., Li H.Q., Cheng Z.J., Wang Y., Zhang X.Y. (2022). Study in genetic diversity of *Gastrodia elata*. Hubei Agric. Sci..

[38] Jiang Y., Hu X., Yuan Y., Guo X., Chase M.W., Ge S., Li J., Fu J., Li K., Hao M. (2022). The *Gastrodia menghaiensis* (Orchidaceae) genome provides new insights of orchid mycorrhizal interactions. BMC Plant Biol..

[39] Kim Y.K., Jo S., Cheon S.H., Hong J.R., Kim K.J. (2023). Ancient horizontal gene transfers from plastome to mitogenome of a nonphotosynthetic orchid, *Gastrodia pubilabiata* (Epidendroideae, Orchidaceae). Int. J. Mol. Sci..

[40] Jin J., Yu W., Yang J., Song Y., de Pamphilis C.W., Yi T.-S., Li D.-Z. (2020). GetOrganelle: A fast and versatile toolkit for accurate de novo assembly of organelle genomes. Genome Biol..

[41] Shi L., Chen H., Jiang M., Wang L., Wu X., Huang L., Liu C. (2019). CPGAVAS2, an integrated plastome sequence annotator and analyzer. Nucleic Acids Res..

[42] Firtina C., Kim J.S., Alser M., Senol Cali D., Cicek A.E., Alkan C., Mutlu O. (2020). Apollo: A sequencing-technology-independent, scalable and accurate assembly polishing algorithm. Bioinformatics.

[43] Zheng S., Poczai P., Hyvönen J., Tang J., Amiryousefi A. (2020). Chloroplot: An online program for the versatile plotting of organelle genomes. Front. Genet..

[44] Benson G. (1999). Tandem repeats finder: A program to analyze DNA sequences. Nucleic Acids Res..

[45] Stefan K., Jomuna V.C., Enno O., Chris S., Jens S., Robert G. (2001). REPuter: The manifold applications of repeat analysis on a genomic scale. Nucleic Acids Res..

[46] Beier S., Thiel T., Münch T., Scholz U., Mascher M., Valencia A. (2017). MISA-web: A web server for microsatellite prediction. Bioinformatics.

[47] Zhang D., Gao F., Jakovlić I., Zou H., Zhang J., Li W.X., Wang G.T. (2019). PhyloSuite: An integrated and scalable desktop platform for streamlined molecular sequence data management and evolutionary phylogenetics studies. Mol. Ecol. Resour..

[48] Katoh K., Rozewicki J., Yamada K.D. (2019). MAFFT online service: Multiple sequence alignment, interactive sequence choice and visualization. Brief. Bioinform..

[49] Lam T.N., Heiko A.S., Arndt V.H., Bui Q.M. (2014). IQ-TREE: A fast and effective stochastic algorithm for estimating maximum-likelihood phylogenies. Mol. Biol. Evol..

[50] Sudhir K., Glen S., Michael L., Christina K., Koichiro T. (2018). MEGA X: Molecular evolutionary genetics analysis across computing platforms. Mol. Biol. Evol..

[51] Jia M., Wang J., Cao D., Jiang C., Li W., Tembrock L.R., Xing G., Li S., Wu Z. (2024). The pan-plastome of *Hemerocallis citrina* reveals new insights into the genetic diversity and cultivation history of an economically important food plant. BMC Plant Biol..

[52] Rozas J., Ferrer-Mata A., Sánchez-DelBarrio J.C., Guirao-Rico S., Librado P., Ramos-Onsins S.E., Sánchez-Gracia A. (2017). DnaSP 6: DNA sequence polymorphism analysis of large data sets. Mol. Biol. Evol..

[53] Camacho C., Coulouris G., Avagyan V., Ma N., Papadopoulos J., Bealer K., Madden T.L. (2009). BLAST+: Architecture and applications. BMC Bioinform..

